# Mosaic Accumulation of Somatic Genetic Variation and Estimates of Age in the Long‐Lived Reef‐Building Coral *Acropora palmata*

**DOI:** 10.1111/mec.70580

**Published:** 2026-10-02

**Authors:** Trinity Conn, Jessie Renton, Valerie F. Chamberland, Zoe Dellaert, Thorsten B. H. Reusch, Benjamin Werner, Iliana B. Baums

**Affiliations:** ^1^ Department of Biology The Pennsylvania State University University Park Pennsylvania USA; ^2^ Conservation Research Department John G. Shedd Aquarium Chicago Illinois USA; ^3^ Barts Cancer Institute, Queen Mary University of London London UK; ^4^ SECORE International Miami Florida USA; ^5^ CARMABI Foundation Willemstad Curaçao; ^6^ Department of Freshwater and Marine Ecology, Institute for Biodiversity and Ecosystem Dynamics University of Amsterdam Amsterdam the Netherlands; ^7^ GEOMAR Helmholtz‐Centre for Ocean Research Kiel, Marine Evolutionary Ecology Kiel Germany; ^8^ Helmholtz‐Institute for Functional Marine Biodiversity at the University of Oldenburg [HIFMB] Oldenburg Germany; ^9^ Alfred Wegener Institute Helmholtz‐Centre for Polar and Marine Research [AWI] Bremerhaven Germany; ^10^ Institute for Chemistry and Biology of the Marine Environment (ICBM), School of Mathematics and Science Carl von Ossietzky Universität Oldenburg Oldenburg Germany

**Keywords:** age, allele frequency, corals, molecular clock, purifying selection, somatic mutations

## Abstract

Somatic genetic variation (SoGV) accumulates during an organism's lifetime and is traditionally viewed as detrimental rather than adaptive due to links with cancer and senescence. However, in modular organisms like corals, deleterious mutations can be purged at the cellular or polyp level, while adaptive mutations may rise in frequency as polyps create genetically distinct modules. Quantifying the somatic genetic landscape in corals is necessary to understand the role these mutations may have in coral and clonal animal development and evolution. Here, we catalogue somatic genetic variation in eight *Acropora palmata* colonies from Curaçao. Whole genomes were sequenced (70–100× depth) to document mutation variant allele frequency shifts as genets age. Large numbers of SoGVs were observed in six‐ to ten‐year‐old colonies, and inferred mutation rates were used to age a genet of uncertain age to almost a century old. Mutations were not fixed at the polyp or branch levels, i.e., they always displayed frequencies < 0.5 as expected at mutating homozygous sites. However, their allele frequencies followed a power‐law distribution, similar to aging human tissues. No signs of positive selection were found; instead SoGVs were under purifying selection. In one colony, mutations in 28 samples from along a branch were analysed using a SNP microarray. Contrary to expectations, genetic and physical distances were unrelated. Along with an observed lack of fixation this pattern may be explained by a large stem cell population, the de‐differentiation or dormancy of stem cells, the contribution of strong purifying selection, or a combination of the previously mentioned. Our findings provide a neutral framework against which to test for module‐level selection of genetic variation in corals, explore the relationship between physical and genetic distance within a colony, and apply a somatic genetic clock to colonies of *Acropora palmata* . This work provides fundamental insights into the landscape of somatic mutations in reef‐building coral, highlighting the importance of studying somatic genetic variation as they may contribute to genetic diversity and adaptability in colonial animals.

## 1 Introduction

Organisms with a modular Bauplan are widespread across the tree of life (Reusch et al. 2021). Clonal species are special modular organisms that consist of a series of repeating modules that can achieve physiological independence and form a population of asexual ramets derived from a sexually produced clone or genet. The modular structure allows for the accumulation and segregation of somatic mutations with time without putting the survival of the colony or genet at risk, because death at the ramet level does not kill the genet. In many modular taxa, somatic mutations can be inherited across sexual generations, including all plants and some early branching animals (Antolin and Strobeck 1985; Vasquez Kuntz et al. 2022) and thus present an understudied source of genetic diversity within modular organisms.

In long‐lived reef‐building corals, the description of somatic genetic variation is relevant not only for studying cellular and evolutionary processes, but also for understanding demographic and ecological processes. Studies of somatic genetic variation can inform researchers, coral restoration practitioners, and marine resource managers about the life history traits and phenotypic variation relevant to restoration and conservation, particularly in corals and other organisms where long lifespans, clonal expansion, and limited genomic resources may have previously prevented such research (Devlin‐Durante et al. 2016; Hughes and Jackson 1980). Further, such studies help connect demographic processes with evolutionary dynamics in novel ways by expanding our understanding of the role of genetic variation within modules to adaptation and genetic diversity at the population level (Ranade et al. 2015; Reusch et al. 2021; Schoen and Schultz 2019).

Previously, a stepwise mutation model of neutral somatic mutation accumulation based on microsatellite markers to age genets was first applied in aspens (Ally et al. 2010) and then corals (Devlin‐Durante et al. 2016). The maximum age detected in the focal species of this study, *A. palmata* , was 838–6500 years depending on mutation rate, indicating that coral genets can be very long lived. With the advent of next generation sequencing, a more accurate somatic clock could be developed. This was first achieved in seagrasses, where a novel universal clock for modular organisms was based on genome wide somatic genetic variants (SoGV) that accumulate and segregate among ramets of a genet of *Zostera marina* through a process of somatic genetic drift (Yu et al. 2020). The central result was that fixed somatic mutations increase linearly with time, thus behaving in a clock‐like manner (Yu et al. 2024). Applied to seagrass clones, the model predicted age in extremely old genets of *Zostera marina* (Yu et al. 2024). This model represents an improvement on previous coalescent models, increasing accuracy of age estimates in long‐lived clonal organisms but has not yet been tested in corals.

The Yu et al. somatic genetic clock was developed using mutations identified as fixed from the allele frequency spectra. However, not all clonal, modular organisms may contain fixed somatic mutations. Before becoming fixed, all somatic mutations start out at low frequencies within a diploid organism. During this stage, genetically distinct cell lineages coexist, creating a genetic mosaic characterized by a wide distribution of somatic mutation frequencies. The fate of these somatic genetic variants varies. They can either be lost from the population, experience positive selection, or be neutral and shift in allele frequency due to genetic drift, including possible fixation or extinction. Previous work in human cancer and cell biology has identified that in normal cells, the Variant Allele Frequency (VAF) spectra of populations of cells under neutral conditions and constant population size approach a 1/f power law distribution, while those experiencing exponential population expansion follow a 1/f^2^ distribution (Moeller et al. 2024). Moreover, neutrality can be assessed by observing the functional impact of somatic mutations across the genome, where neutral forces should create a pattern of random distribution of mutations across the genome. Deviations from these expectations may indicate positive selection and inform about the size of the stem cell pool present. This is because the likelihood of fixation of neutral variants increases with decreasing size of the stem cell pool that forms new modules. In plants, including seagrasses, this stem cell pool is usually small [3–4 cells (Burian et al. 2016; Yu et al. 2024)] while the stem cell pool size contributing to the generation of a new coral polyp is unknown. A large stem cell pool forming new modules may prevent fixation of somatic mutations even in long‐lived genets as it is less susceptible to random somatic genetic drift. In such a case, it is not clear if the Yu et al. somatic genetic clock can still be used to age genets.

As modular organisms grow and accumulate modules over time, one would expect that the accumulation and segregation of somatic mutations would increase with spatial distance relative to the founding module. In corals, the situation is more complex than in simple modular organisms such as seagrasses or other rhizomatous clonal plants. Coral modules are produced at two levels, the polyp and colony fragment level. Either level can give rise to entire daughter colonies (Highsmith 1982). First, the colony grows through the asexual division and propagation of polyps within a colony. These polyps are the modules relevant to the modelling of the accumulation of somatic mutations—they (usually) can survive on their own when separated from the colony and they are formed by independent divisions, either intratentacularly or extratentacularly (Coates and Jackson 1987). When embedded in a colony, polyps share resources and signals through a thin layer of tissue (Oren et al. 2001). These polyps can then continue to produce daughter polyps through polyp divisions (Figure 1a), which will accumulate SoGV over time, generating a diverse genetic landscape across a colony of a scleractinian coral. Studies at the polyp level thus provide insights into the dynamics of allele frequency changes within genets.

**FIGURE 1:**
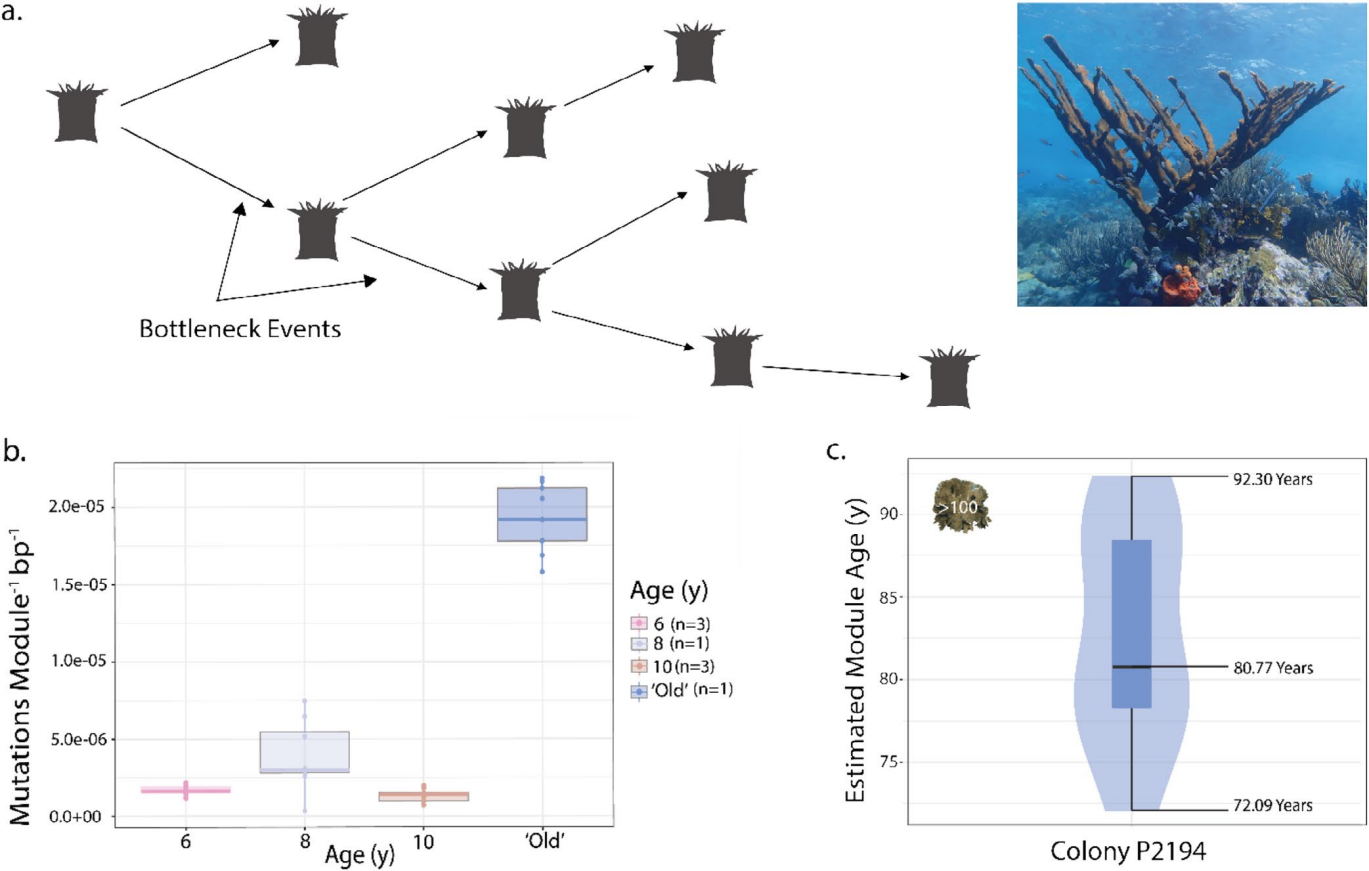
The accumulation of somatic mutations in a colony of *Acropora palmata* to estimate the age of a module. (a) The process by which modules within a genet of *A. palmata* can differentiate over the lifetime of the colony. Each time a module (polyp) divides, a subset of cells within a polyp are recruited to contribute to the new module. This results in a bottleneck in the genetic diversity that resulted from mutations within the modules. Overtime, as mutations accumulate in the dividing polyps, modules eventually become genetically distinct, and the pairwise genetic distance between them increases. Polyp division can result in large, old *A. palmata* colonies consisting of hundreds of thousands of polyps as pictured. (Photo: Zoe Dellaert). (b) Mutation rate per module was calculated for each age class (6, 8, 10 years old and a colony of uncertain age). Each boxplot shows the median (dark‐coloured line in the central portion of distribution) and the ranges (points) of the mutation rates calculated for all modules within an age class. The top and bottom of the boxes represent the 25% and 75% quartiles of the range of values. (c) Distribution of estimated ages based on rate of SoGV occurrence in modules within the ‘old’ colony of uncertain age. The youngest estimated age was 72.09 years old, while the oldest estimated age was 92.30 years old. True genet age likely falls closer to the median of these estimates, approximately 80.77 years old. This is younger than the previously estimated age of this genet using microsatellites, 228–1194 years old (Devlin‐Durante et al. 2016).

An ecological extension of this work is to provide much needed accurate estimates of genet age for demographic models. Occasionally, physical disturbance results in the fragmentation of colonies, forming physically independent ramets of a single genet (Baums et al. 2006; Lirman 2000). Therefore, a small colony may stem from a large old genet or from a recent sexual recruit. Previous attempts at aging have used size, and skeleton cores to date the age of a coral (Tomiak et al. 2016). However, aging the skeleton only informs us about when the skeleton of that ramet (colony) was produced and not necessarily when the genet first recruited into the population (i.e., genet age) prior to any fragmentation events (Irwin et al. 2017). Our difficulties with aging genets also mean that we lack crucial information on population dynamics of reef building coral species such as the frequency of sexual recruitment which makes it more challenging to manage these species. Here, we had access to colonies of known age that resulted from sexual coral restoration efforts, a prerequisite to solving the problem of aging coral genets.

In this study, the aim was to generate a clock that assesses the genet age structure of existing populations of *Acropora palmata* , currently missing from population management toolkits. Whether the dynamics of SoGV in corals behave more like modular plants (seagrasses) or other animals (like humans) is still unknown. Here, we look to these two systems to understand whether somatic mutations within coral colonies are shaped primarily by drift or selection. If corals behave like seagrasses, stem cell pool (N_e_) and mutation rate are two of the most important factors influencing the allele frequencies of mutations in the population of modules (Yu et al. 2024), generating insights into the stem cell biology of these species in addition to aging genets (Moeller et al. 2024). We use deep sequencing of colonies of known age to test the applicability of a previously developed universal somatic genetic clock (Yu et al. 2024) in corals, quantify the allele frequency spectra of somatic mutations in corals and identify signals of selection or neutrality. We describe three major aspects of the role of somatic genetic variation in corals: (i) the somatic genetic landscape in *Acropora palmata* , (ii) the correlation between physical proximity of polyps and shared SoGV, and (iii) the applicability of a somatic genetic clock through calibration with colonies of known age.

## 2 Materials and Methods

### 2.1 Sample and Data Collection

Seven *Acropora palmata* colonies of known age were selected from a cohort of outplants monitored by SECORE across two reef sites in Curaçcao (Sea Aquarium: 12.0839146°N, 68.894431°W; Marriott: 12.11888613°N, −68.9685206°W; Table S1). Colonies represented three age classes: 6 years old (*n* = 3 colonies), 8 years old (*n =* 1 colony), and 10 years old (*n* = 3 colonies). These colonies are part of cohorts produced under human care during coral spawning in 2011 and 2015. Scientists at SECORE International and CARMABI have monitored these colonies since they were outplanted. These colonies were previously genotyped using the Applied Biosystems Axiom Coral Genotyping Array—550962 (Thermo Fisher, Santa Clarita, CA, USA) (Kitchen et al. 2020).

To quantify intra‐colonial somatic heterogeneity, each colony was sampled 5–6 times using coral cutters. Samples consisted of 3–4 polyps at each sampling site and the location of each sample was documented both with images and in the sampling notes. These samples were considered modules of the colony. Samples from the 6‐year‐old colonies and the 10‐year‐old colonies were collected in December of 2021, while samples from the 8‐year‐old colony were collected in September of 2019. Intra‐colonial samples (*n* = 9) were also collected from one ‘old’ colony previously estimated to be 228–1924 years old using microsatellite markers (Devlin‐Durante et al. 2016). Samples were preserved in 95% ethanol and kept at room temperature until return to Penn State University and then transferred to −20°C. DNA was extracted using the Qiagen DNeasy Kit (Qiagen, MA), following the modified protocol by Kitchen et al. (2020) (https://doi.org/10.17504/protocols.io.bgjqjumw). All libraries were prepared following the Truseq PCR‐Free protocol (Illumina, CA). Samples were sent for paired‐end whole genome sequencing with an insert size of 150 bp and a target depth of 80× on the NovaSeq 6000 (Table S1).

Sample collection and shipping complied with local and international laws as outlined in the permit section. Impact on natural populations was minimal as sample sizes were very small (3–4 polyps). No experiments on live animals were conducted, nor were animals kept in a laboratory.

### 2.2 Data Processing

Sequencing data was checked for quality using FastQC version 0.11.8 (https://www.bioinformatics.babraham.ac.uk/projects/fastqc/). Sequences were trimmed for quality and adaptor contamination using BBduk version 39.06‐1 (https://jgi.doe.gov/data‐and‐tools/bbtools/bb‐tools‐user‐guide/bbduk‐guide/). The following parameters were used to trim the sequences: (trimq = 20, minlen = 50, maq = 20). Sequences were aligned to the *Acropora palmata* genome (Locatelli et al. 2023) with the Burrows Wheeler method using BWA‐MEM v0.7.17 under default parameters (Li and Durbin 2009). Reads were then compressed and sorted using SAMTools version 1.20.0. Duplicated reads were marked and removed using the MarkDuplicates tools in GATK version 4.0.1.2. Properly paired reads with a minimum mapping score of 20 (−q 20) were retained using SAMtools version 1.20.0 (Danecek et al. 2021). Coverage across scaffolds was calculated using bbmap version 39.06‐1 (Table S1).

### 2.3 Identification of Somatic Mutations

Somatic mutations were identified using Mutect2 in GATK version 4.0.1.12 using standard settings and the *Acropora palmata* reference genome (Benjamin et al. 2019) using a tumour‐normal pairing‐based method. All samples within a genet were compared in a pairwise fashion, with each sample treated as the ‘tumour’ sample and the ‘normal’ sample within the Mutect2 syntax. Identified SNPs (single nucleotide polymorphisms) were then filtered following the standard parameters set out by Mutect2. Mutect2 is able to detect mutations at frequencies as low as 0.01 within the data. Also, only SNPs with a mapping quality of 20 or greater and a minimum base quality of 25 or greater were kept. The total number of SoGVs for each sample within a genet was determined as the sum of all unique mutations discovered for a sample when the sample was classified as the ‘tumour’ in the pairwise comparison. Mutations were filtered for a depth of 60 or greater using a custom script in R (vrf50.R).

### 2.4 Calculating Substitution Burden

Variant call files (VCFs) containing identified mutations were exported using bcftools version 1.20‐0 (Danecek et al. 2021) and loaded into R v.4.3.2. Using a custom R script (vrf50.R), the mutation load for each sample within a genet was determined as the sum of all unique mutations discovered for a sample when the sample was classified as the ‘tumour’ in the pairwise comparison. Mutations that were found in a ‘tumour’ sample in more than one comparison were collapsed to one occurrence when estimating substitution burden.

We used the total unique mutation load per module (a physically distinct portion of the colony represented by each sample that was taken) per callable bases in the genome for that module to estimate a mutation rate, as instructed by best practices (Bergeron et al. 2022). Callable bases were calculated as the total bases covered in the output of bbmap. Mutation rate was calculated as:

$$\text{MutationRate}\;{\text{module}}^{-1}{\mathrm{bp}}^{-1}=\frac{\text{TotalUniqueMutationsPerModule}}{\text{CallableBases}\in \text{Genome}\;\left(\mathrm{bp}\right)}$$

### 2.5 Estimation of Age

The estimated mutation rate described above was used to calibrate a modified somatic molecular clock to age the modules of the genet of uncertain age, building on previous work by Yu et al. (2024). A mutation rate per module was estimated as the mutation rate year^−1^ in the colonies of known age. The mean mutation rate of occurrence per year was then applied to calculate the age of each module in the genet of uncertain age (Figure 1c). Age of each module within genet P2194 was calculated as follows:

$$\mathrm{Age}\;\text{of Module}\;\left(\text{years}\right)=\frac{\text{Rate of Mutation Occurence}}{\text{Year}\;}*\text{Total Mutation Occurence}\in \text{Module}$$

### 2.6 Distribution of Mutations Across Modules

To investigate how mutations segregate within a colony, the distribution of shared mutations was analysed. Mutations were classified as ‘private’ if that mutation was not identified in any other sample. Mutations were classified as ‘shared’ if that mutation was identified in greater than one sample for samples treated as tumours. The assumption is that it is more likely that these mutations were shared through shared ancestry rather than the same mutation occurring independently in two tissue samples. A presence/absence matrix of all identified mutations per module was created in R and intersections of mutations were calculated using the R package UpsetR (Conway et al. 2017). Mutations were displayed using an UpSet plot that shows the relationship of the mutations across a sampled colony through displaying all intersection sets between modules sampled. Identity and allele frequencies of shared and private mutations were extracted using a custom R script (vrf50.R) and plotted (Figure 4).

### 2.7 Co‐Occurrence Network Analysis

To visualize the relationship between the spatial distribution of genetic diversity (identified somatic mutations) and the physical distance between modules, a co‐occurrence network was built for colony M145 (6 years old). A presence/absence matrix was created for all mutations across each sample. A co‐occurrence matrix was created by multiplying the matrix against itself transposed in R (co_mat<−*t*(presabsmatrix)%*% presabsmatrix). This matrix was then plotted as a network using the R package igraph (Csardi and Nepusz 2006). Edges indicate the degree of relatedness, where shorter edges indicate that the two modules share more mutations, and longer edges indicate that the two modules that share an edge share fewer mutations between the two of them. The size of nodes in the matrices is indicative of the total number of mutations that module shares with the remainder of the colony. Therefore, the smaller the node, the more distinct the module is from the remainder of the colony. The larger the node size, the more similar the module is to the remainder of the colony (Figure 5b).

### 2.8 Detecting Population Processes in Allele Frequencies

Mutation location, reference and alternative bases, and variant allele frequency were exported using bcftools. Using a custom R script (vrf50.R), mutations were filtered by depth and by allele frequency. All calls per genet were consolidated into one dataset. Mutations were then classified as ‘fixed’ or ‘mosaic’. Fixed mutations were defined as mutations identified with a minimum variant allele frequency of 0.5, while the reference allele had a variant allele frequency less than 0.01 (Yu et al. 2024). Any mutations with a variant allele frequency between 0.01 and 0.49 within the sample categorized as the ‘tumour’ sample were categorized as mosaic mutations.

Studies of healthy human somatic cell populations suggest that allele frequencies of cells under neutral processes should demonstrate a relationship of 1/f or 1/f^2^ in a clonal population undergoing a stable phase (1/f) or a growing phase (1/f^2^) under neutral conditions (Moeller et al. 2024). Since no fixed mutations were detected, even in a colony of previously determined high age (via microsatellite genotyping), the clock was instead parameterized using mosaic genetic variation, and a model similar to healthy human tissue, where fixation is limited, was used to assess deviation from neutrality. Therefore, under neutral conditions it is predicted that the allele frequencies of mutations identified in the coral tissue should follow the 1/f^2^ power law distribution (Raine Detmer et al. 2016). To test the deviation of the allele frequency distributions from a neutral distribution, the R package neutralitytestR (Williams et al. 2016) was used. Significance of deviations were assessed by three statistics: the Kolmogorov–Smirnov statistic, the area under the curve (AUC), and a linear regression between the cumulative distribution functions (CDF) of the empirical data and a simulated neutral distribution.

We used the R package MOBSTER (Caravagna et al. 2020) to further detect whether there were any signals of individual subclonal clusters (genetically distinct groups of sampled cells within a module). These clusters may represent distinct populations that are the result of genetic drift and selection favouring the propagation of the descendants of a single cell. MOBSTER was designed to identify patterns of clonal expansion in bulk sequencing of cancer cells and can identify signals of positive selection as well as capture the neutral evolutionary forces that shape the allele frequency spectra within cancer cell populations (Caravagna et al. 2020). MOBSTER uses a finite mixture model with mixed distributions to identify clonal expansions and neutral dynamics with a 1/f^2^ tail. The model then determines the optimal *k* to suggest if a sample is monoclonal or polyclonal (Caravagna et al. 2020).

### 2.9 Annotating Identified Variants

To annotate any functional impact of somatic mutations discovered, the R package dndscv (Martincorena et al. 2017) was used. The functional impact of all mutations located in coding regions was established as either a synonymous mutation, a nonsense mutation, or a missense mutation. Under neutrality, the proportion of mutations identified in coding regions should be statistically like the proportion of the entire genome that is made up of coding sequences. A chi‐square test was performed to determine if the observed proportion of mutations discovered in coding sequences was less or more than expected under neutral conditions. All statistical analyses were performed using R v.4.3.2.

### 2.10 Analysing Spatial Distribution of Somatic Mutations Using a SNP Array

#### 2.10.1 Sample and Data Collection

In 2019, one large branch of a colony in Curacao was sampled (Blue Bay, 12.13492, −68.9871) (Table S2). A transect was placed along the branch, and samples were taken every 10 cm along the transect. Samples were also taken at the tips at the end of the branch (Figure 5a,b). These samples were preserved in 95% Ethanol for processing on the StagDB array. DNA was extracted using the Qiagen DNeasy Kit (Qiagen, MA) (https://doi.org/10.17504/protocols.io.bgjqjumw) and sent to ThermoFisher for genotyping on the Applied Biosystems Axiom Coral Genotyping Array—550962 (Thermo Fisher, Santa Clarita, CA, USA).

#### 2.10.2 Data Processing SNP Array

Raw data from the SNP array were analysed using the Axiom ‘Best Practices Workflow’ using the default settings. Settings were set so that the sample Dish QC > 0.82, plate QC call rate > 97, SNP call‐rate cutoff > 97, and the percentage of passing samples > 95. Resulting genotyping files were converted to variant caller format (VCF) using the bcftools plugin affy2vcf (https://github.com/freeseek/gtc2vcf). The VCF was loaded into R v.4.3.2 for downstream analyses.

#### 2.10.3 Identification of Somatic Mutations Using a SNP Array

All probes with missing data were removed from analysis. Loci with somatic mutations were identified as any locus where not all samples within the genet carried the same allele call at that site. This is consistent with previous assays of somatic mutations in *Acropora palmata* using a SNP array (Vasquez Kuntz et al. 2022).

#### 2.10.4 Genetic and Distance Correlation

To test if the accumulation of somatic mutations followed expectations that genetic and physical distance should correlate (Gates et al. 2019; Liu et al. 2024; Slatkin 1993), two methods were used. First, based on the scaled images taken during sampling, the distance between samples along the transect was determined. Prevosti's genetic distance was calculated between all samples using the R package poppr (Kamvar et al. 2014), derived from the set of probes identified to contain mutations. A linear regression was run between genetic and physical distance using R v4.3.2.

## 3 Results

### 3.1 Calibrated Substitution Burden to Estimate the Age of a Genet of *Acropora palmata*

To first understand the overall contribution of somatic mutations to the genetic diversity of *Acropora palmata* , the rate of SoGV accumulation was calculated as the rate of occurrence of mutations per module per base pair (Table 1 and Table S3). The mean substitution rate of detected mutations across all aged colonies equalled 2.798e^−7^ ± 1.977e^−7^ mutations bp^−1^ module^−1^ year^−1^.

**TABLE 1:** Average substitution rate across *A. palmata* genet age classes.

Age of genet (year)	Average substitution rate (mutations bp^−1^ module^−1^)	± SE (mutations bp^−1^ module^−1^)
Six	1.687e^−6^	3.227e^−7^
Eight	3.854e^−6^	2.327e^−6^
Ten	1.313e^−6^	3.900e^−7^
Uncertain (> 100)	1.919e^−5^	2.225e^−6^

Using the mean substitution burden across colonies of known age, the age of the modules across the old colony of uncertain age was estimated and ranged from 72.09 to 92.30 years (Figure 1c), markedly lower than previous estimates using microsatellites of 228–1924 years (Devlin‐Durante et al. 2016).

### 3.2 Distribution of Variant Allele Frequencies

Under the given filtering conditions, none of the detectable SoGVs were fixed (Figure S1). Fixed SoGV were defined as those with a read depth of at least 60×, a variant allele frequency of at least 0.50, and a normal (reference) allele frequency of 0.01. Although previous work has established the use of the variance around the fixed allele frequency of 0.5 (Yu et al. 2024), given no mutations were found at a frequency higher than 0.5, this method was deemed inappropriate in this study and an alternative approach was developed that utilised mean substitution burdens (see preceding section). Nevertheless, allele frequency distributions changed with age as follows.

First, allele frequency distributions within genets were deconvoluted using MOBSTER to study the effects of drift and identify any putative subclones that have expanded within genets due to selection. If allele frequency distributions were mostly shaped by drift, we expected a higher frequency cluster of mutations that became fixed over time and a low frequency tail of recent mutations. Under selection, a third cluster at mid‐frequency would be expected, containing alleles with a fitness advantage accompanied by hitchhiking neutral variants. The best model as determined by Integrated Classification Likelihood (ICL) and/or Akaike Information Criterion (AIC) detected two clusters present in all genets. These clusters represent the low frequency mutations and mutations that have risen to higher frequency. The mean of Cluster 1, higher frequency variants, was highest in the old colony (0.25) (Figure 2). This analysis confirmed the lack of a detectable third cluster which would have indicated the presence of a subclone under selection.

**FIGURE 2:**
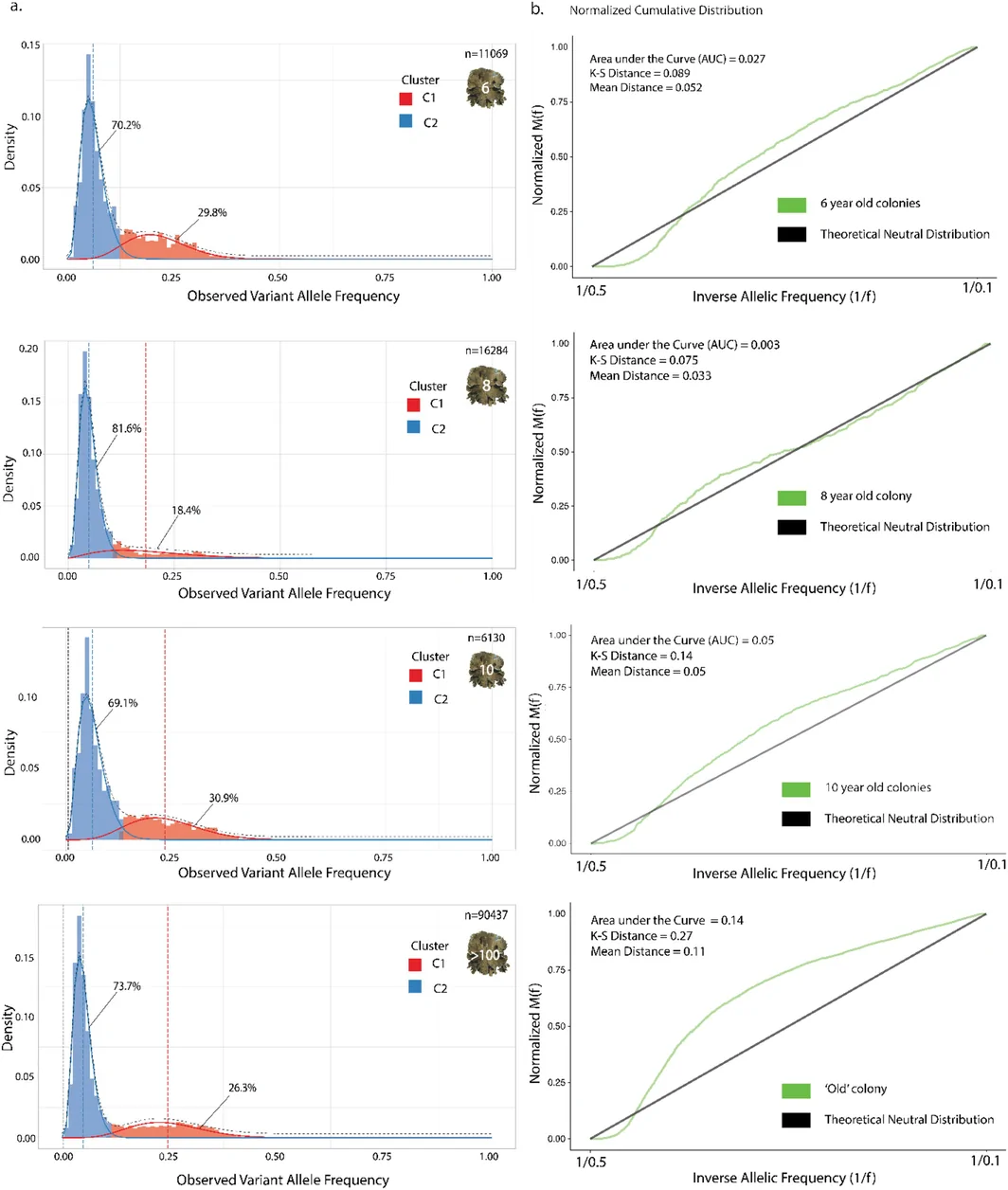
Population dynamics of somatic mutations in *A. palmata* . No clear signal of positive selection was detected across all four colony age classes analysed in this study. (a) Cluster deconvolution of allele frequency spectra across all four colony age classes. MOBSTER detected two subclones per colony regardless of age. Each colony was made up of mostly polyclonal clusters at low allele frequencies, with one subclone rising to higher allele frequencies. Cluster 1 within each age class is in red, and cluster 2 within each age class is in blue. Dotted line of each cluster's colour represents the mean variant allele frequency of that cluster. (b) Identifying signals of neutrality in variant allele spectra. Each plot represents an age class. Black line is the normalized cumulative distribution function of the theoretical 1/f neutral distribution. The green line within each plot depicts the normalized cumulative distribution function of the empirical allele frequency within each age class. Statistics of significance for each age class are listed within each plot for each age class.

The variant allele spectra of the colonies within the three classes of known age (six, eight, ten years old) did not diverge significantly from the expected neutral 1/f distribution (AUC = 0.0273; 0.0029; 0.0584, K‐S Distance = 0.0895; 0.0751; 0.1314, Mean Distance between CDFs = 0.0519; 0.0337; 0.0524, *R* ^2^ = 0.9790; 0.9886; 0.9872) (Figure 2). In contrast, the variant allele spectra of the modules within the ‘old’ colony did significantly diverge from the neutral 1/f distribution (AUC = 0.1423, K‐S Distance = 0.2671, Mean Distance between CDFs = 0.1144, *R* ^2^ = 0.9581). Here, the distribution was closer to 1/f^2^ at higher allele frequencies (Figure 2), possibly evident of other demographic processes or selection with age possibly resembling clonal expansions in aging human tissues (Mon Père et al. 2026), although more evidence is needed to make further conclusions.

While there was no fixation of somatic mutations across all colonies analysed, the density of mutations found in the mosaic region (allele frequency of 0.1–0.5) increased with age (Figure 3). Comparisons of the number of mutations in the mosaic region across all age classes were significantly different (*p* < 0.01) (Figure 3b) and the calculated K‐S distance value increased with age (Figure 3b) in a non‐parametric two‐way Kolmogorov Smirnov test. Further, with increasing age, the density of mutations found at higher allele frequencies significantly increased (Kolmogorov–Smirnov, *p* < 0.05) (Figure 3).

**FIGURE 3:**
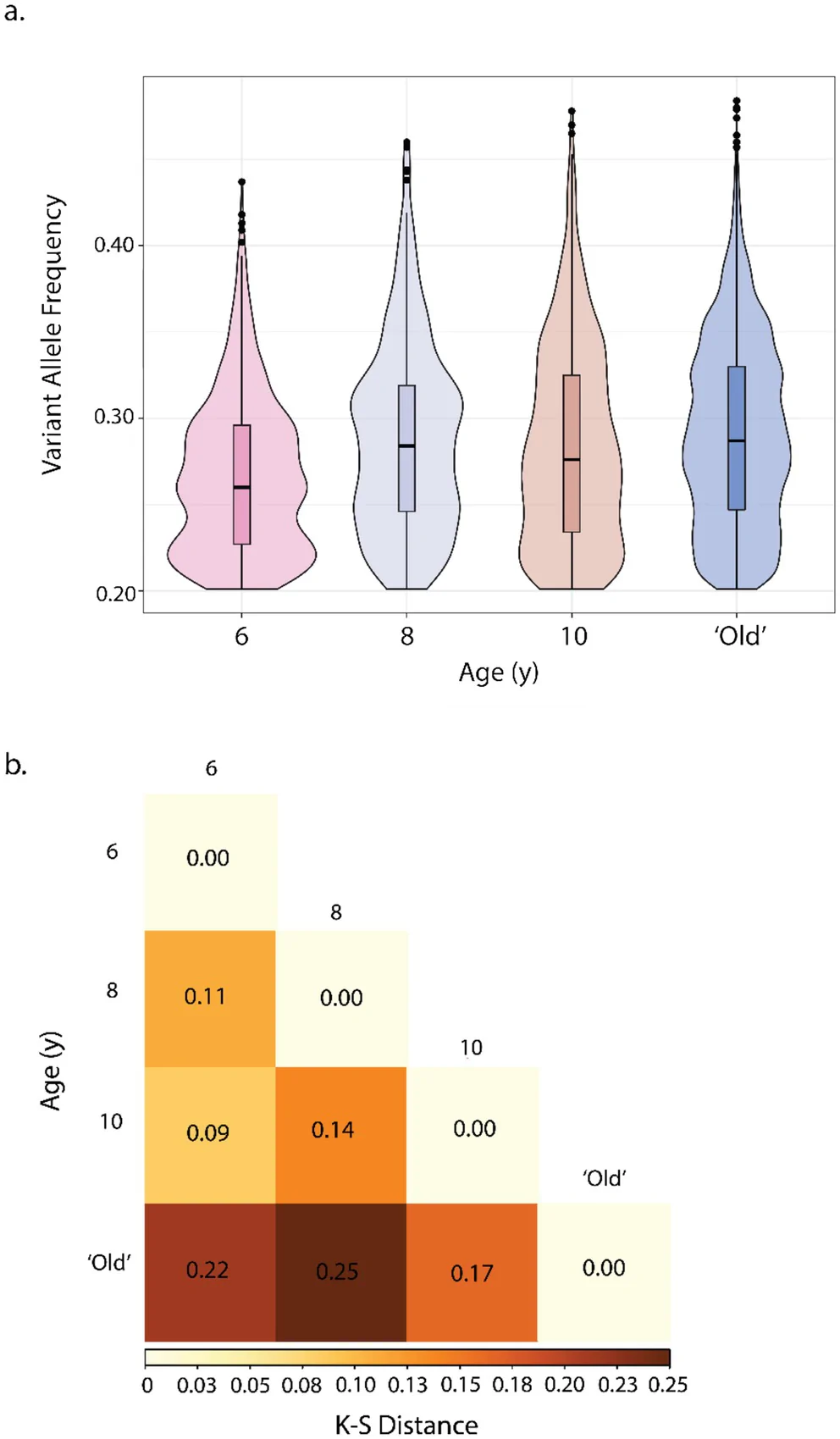
Shifts in allele frequency with age in *Acropora palmata* . (a) Violin plot of mosaic mutation variant allele frequencies in *A. palmata* colony age classes. Density of mutations at higher frequency increased with age, with the highest density of mutations at high frequency in the ‘old’ colony of uncertain age (blue). Only allele frequencies between 0.2 and 0.5 are shown. The boxplots within the violin plots show the median, the range of allele frequencies, and the 25% and 75 quartiles. Width of violin plot emphasizes distribution of allele frequencies within these quartiles. (b) Kolmogorov–Smirnov distances of all pairwise comparisons between allele frequency distributions from different age classes shown in (a). All comparisons were significantly different (*p* < 0.05). Darker coloured squares indicate a greater K–S distance. The Kolmogorov–Smirnov test statistic is the maximum distance between the cumulative distribution functions of the two distributions being compared.

### 3.3 Homogeneity of Genetic Diversity Within a Colony of *Acropora palmata*

The majority of identified SoGV were private alleles. Mutations were shared among multiple modules in all colonies except for colony S15 (Table S1). Allele frequencies across intersection sets shifted towards higher frequency mutations with more intersection sets (Figure 4c). A co‐occurrence network of mutations in colony M145 (Figure 4b) confirms this analysis, indicating that some modules are more homogeneous with the rest of the population than other modules.

**FIGURE 4:**
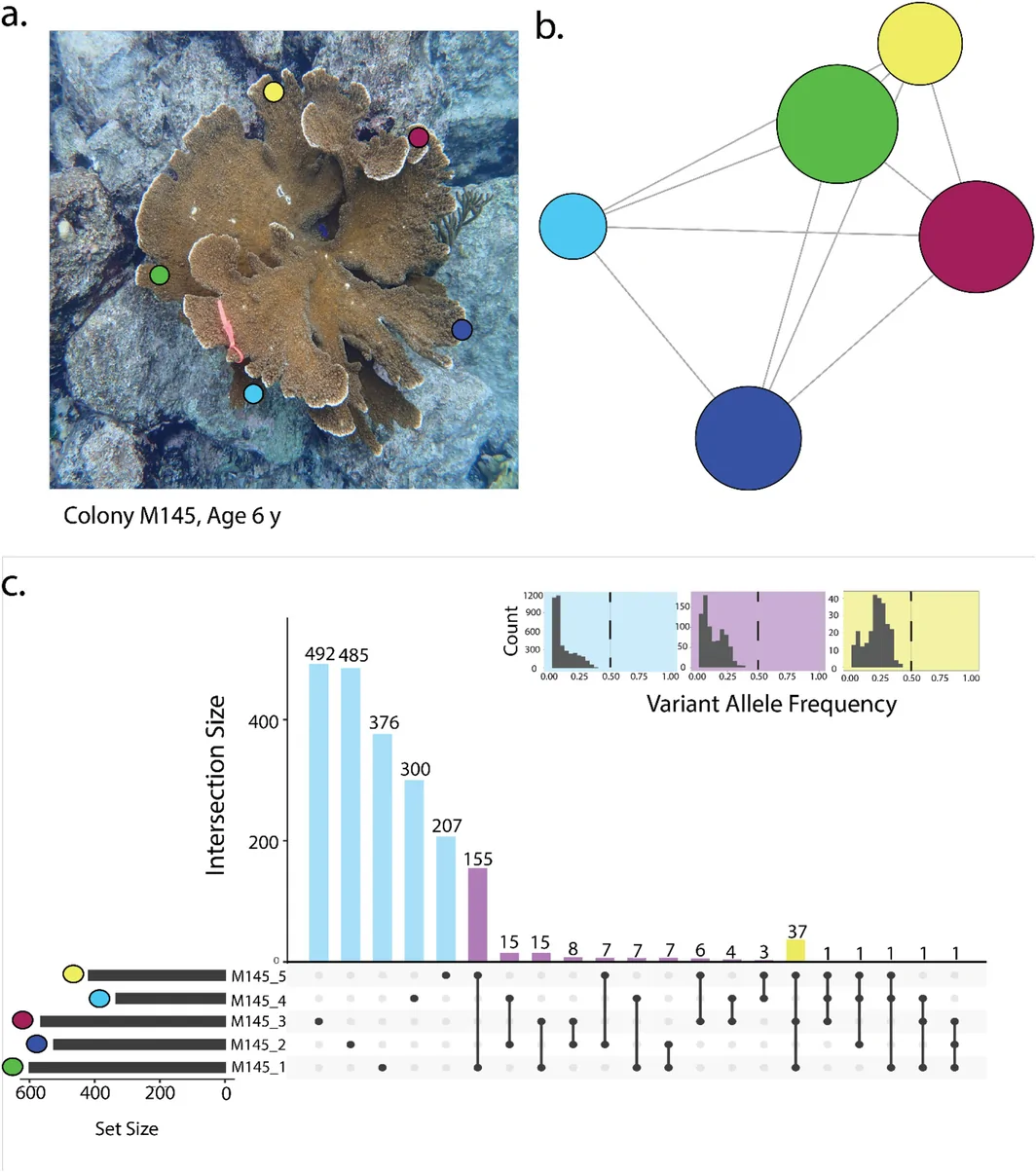
Homogeneity of genetic diversity within a genet of *Acropora palmata*. (a) Colony M145, a 6‐year‐old colony, was selected to display the relationship between the physical distribution of modules within a genet and the distribution of mutations across modules. Photo was taken by Zoe Dellaert. Colony M145 is approximately 60 cm in diameter. Coloured circles on the colony image (green, yellow, maroon, light blue, dark blue) indicate the location of sampled modules analysed in this study. (b) A co‐occurrence network of the mutations displays the connectivity of stem cells within genet M145. The nodes represent the modules, coloured by their sampling location as in (a). Larger nodes indicate higher connectivity with other modules. Edge length is correlated with the number of shared mutations, longer edge lengths indicating fewer mutations are shared between the two modules. Module M145_1 (green) had the highest connectivity with other modules, while the light blue module had the least connectivity with other modules, indicating a more unique evolutionary history. (c) UpSet plot of intersection sets of mutations across all modules within colony M145. Modules are labelled by colour as they were in (a). Intersection sets were categorized as private mutations (light blue bars), mutations shared between two modules (purple bars) and mutations shared with 3+ modules (yellow bars). Allele frequencies of mutations within each category of intersection set were extracted and plotted in the upper right hand of the plot. The background of each plot of observed allele frequencies is colour coded by mutation category. A black dotted line represents a variant allele frequency of 0.5.

### 3.4 Spatial Distribution of Allele Frequencies

The distribution of allele frequencies of private mutations encompasses primarily low frequency variants (Figure 4c and Table S4). The next largest group were mutations shared by two modules (intersection sets). The distribution of allele frequencies of mutations shared between two modules contained both mosaic mutations and low frequency mutations (Table S4). Mutations shared between three or four modules were primarily low frequency and mosaic mutations (Table S4).

We expected that genetically distinct subclones arising during the growth of the colony should be geographically constrained to near the module in which they originated. This expectation was not met in any of the colonies analysed in this study. The majority (> 85%) of identified mutations across all genets were private alleles, only detected in one sample in a colony. Mutations that were shared and retained across regions of the colony are part of higher frequency subclones maintained and propagated across the genet and were also likely generated earlier in the history of the genet, in a shared common cellular ancestor (CCA) of multiple modules. There is an overall shift to higher allele frequencies as mutations are shared with a higher number of modules. However, even low frequency mutations are represented within all intersection sets. Since it is unlikely that the same mutations have arisen in multiple modules independently, some of these mutations were being maintained at low frequency, even after propagation to multiple modules within a genet.

Further evidence of shared SoGV across a colony of *Acropora palmata* regardless of their geographic origin emerged from an analysis of somatic mutations using a different method, a 19,696 SNP microarray. There was no significant linear relationship between genetic and physical distance (*R* ^2^ = 0.0714) along the branch of the colony assayed using the SNP array (Figure 5). There was also discordance between the neighbour joining tree produced by the pairwise genetic distances and the neighbour joining tree produced by the pairwise physical distances. The path‐score difference between these two trees was 19.570.

**FIGURE 5:**
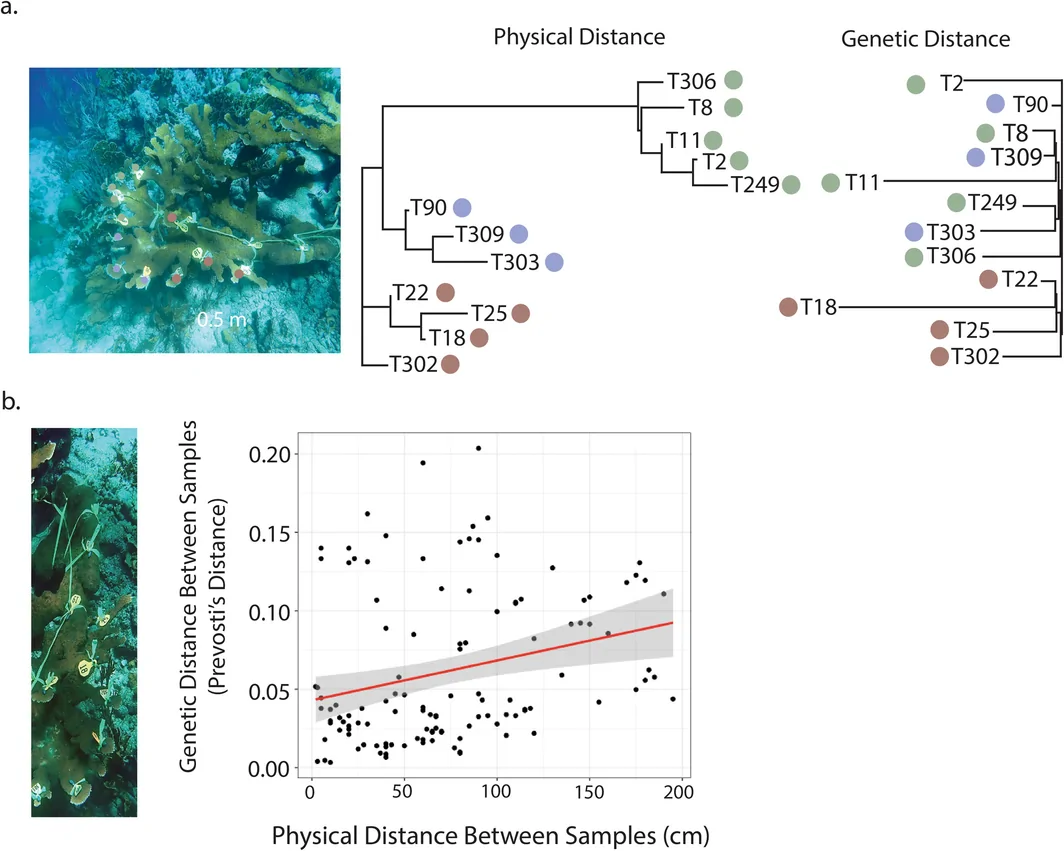
Genetic distance between modules does not correlate with their physical distance along a branch and among the tips of an old *A. palmata* colony. (a) Tips of a branch of *Acropora palmata* show discordance between their physical distance (in cm) and their genetic distance (Prevosti's genetic distance). Sampled tips are flagged in the photo. Shown is a neighbour joining tree built using the pairwise physical distance between the tips of the branch. Green, blue and red dots indicate three major physical branching events within the colony, that resulted in clusters of closely related growing edges. The neighbour joining tree of pairwise Prevosti's genetic distances between the sampled tips lacked a clear branching pattern. Physical distance and genetic distance thus generated different topologies. (b) Linear regression between the Prevosti's genetic distance and the physical distance (in cm) between samples taken along a transect on the same large branch. There was a weak association between the physical distance and the Genetic distance between samples (*R* ^2^ = 0.072, *p* = 0.0031).

### 3.5 Functional Consequences of Somatic Mutation Accumulation

For all sequenced samples with at least one mutation detected in coding regions, there was a significant depletion of mutations in coding regions (*χ* ^2^, *p* < 0.05) (Figure 6). The proportion of mutations discovered in coding regions across all age classes was never greater than 0.0267 (Table S5), significantly reduced relative to the total proportion of coding regions in the entire genome (0.1300).

**FIGURE 6:**
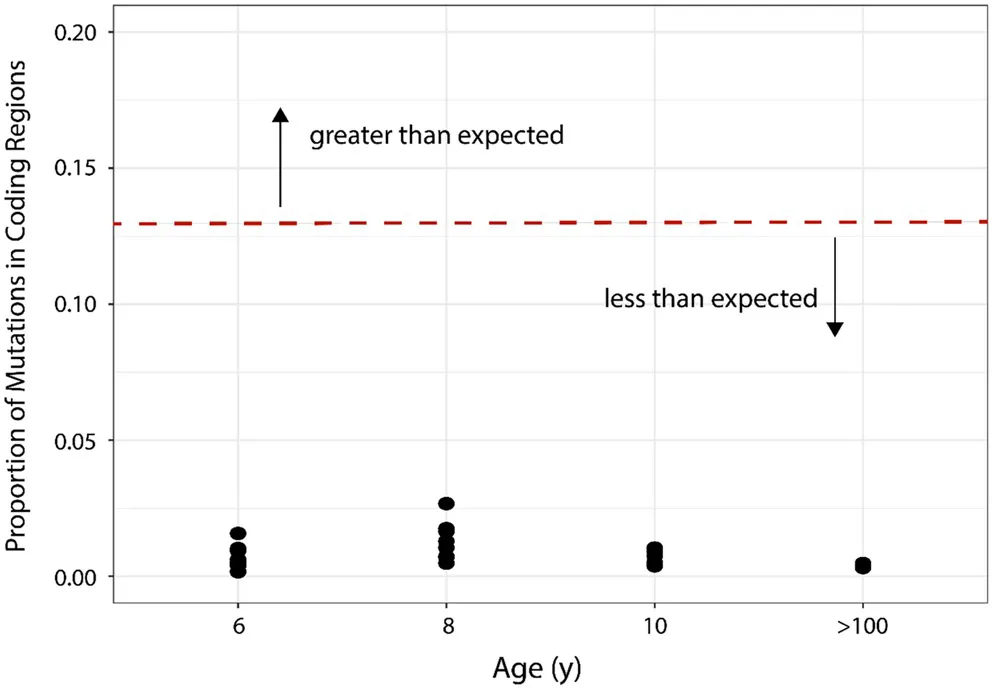
Purifying selection within a genet of *A. palmata* . Scatter plot of proportion of mutations within a module (sample) discovered in coding regions within the *A. palmata* genome across the four age classes tested in this study. Red dotted line is the proportion of coding sequences in the *A. palmata* genome. Modules within all age classes were significantly depleted of mutations in coding regions (*χ* ^2^, *p* < −0.05).

To determine whether high somatic mutation rates were associated with methylation across the coral genome, CpG islands were identified as a proxy for regions of the genome likely experiencing elevated methylation (Data S1). Overall, mutations located in putative CpG islands comprised on average 0.25% of mutations identified. This was consistent across all colonies and age classes, and no significant increase with age was evident in the proportion of mutations recovered from CpG islands (*p* > 0.05, ANOVA) (Figure S2).

## 4 Discussion

Here, we show that somatic genetic variation in eight *Acropora palmata* colonies from Curaçao increased with genet age. Based on the average SoGV accumulation rate of 2.798e^−7^ ± 1.977e^−7^ mutations bp^−1^ module^−1^ year^−1^, we applied a somatic genetic clock to estimate the age of a genet of uncertain age as 80.77 years, somewhat younger than previous estimates that had used a coalescent approach (228–1194 years old). In coral genets as young as six to ten years old, replicate samples sequenced to a depth of at least 80× revealed high somatic genetic variation across the genomes of all genets. Although mutations were not fixed at polyp or branch levels, their allele frequencies followed a power‐law distribution (Figures S3–S7), similar to patterns in aging human tissues, and were not under detectable positive selection. Instead, depletion of mutations in coding regions suggests strong purifying selection, as also seen in Yu et al. (2020). It is likely that high‐fidelity DNA repair mechanisms oppose the high substitution burden in coding regions. Surprisingly, genetic and physical distances were unrelated in samples taken along the growth axis of a large colony. These observations support the hypothesis that corals, in contrast to previous observations in vascular plants, derive new modules from a large stem cell pool, delaying fixation for a very long time. Alternatively, the observed lack of fixation and the disconnect between genetic and physical distances may be explained by multiple stem cell lineages that are shared across the colony. Together, these findings have implications that extend beyond coral molecular biology. Understanding how somatic mutations accumulate and are maintained within coral colonies may improve estimates of population age structure, recruitment history, and adaptive potential in threatened reef systems where long‐term demographic data are limited.

### 4.1 A Somatic Molecular Clock for *Acropora palmata*

The somatic genetic clock developed in this study yielded estimates of genet age markedly lower than microsatellite‐based estimates of genet age. Previous estimates of genet age in coral populations were derived from a coalescent model of somatic mutation accumulation (Devlin‐Durante et al. 2016). The method relied on a limited set of microsatellite markers for which no mutation rate was known and could only be applied to genets with at least five living ramets. Mutation rate estimates were bounded by the geologic record (the maximum age of a genet was set to the emergence of shallow water habitat ~6000 years ago) and the approximate growth rate of the colony with the smallest total genetic divergence (minimum genet age estimate), yielding large uncertainties (Devlin‐Durante et al. 2016). As well, microsatellites can mutate both forward and backward, generating error in the assumptions needed for a somatic genetic clock. The method is therefore skewed towards the higher range of genet ages, and it does not provide information on the population genetic processes affecting somatic mutations within colonies.

Here, we instead modify the somatic genetic clock designed for fixed genetic differences presented in Yu et al. (2024). As no fixation was observed, the somatic genetic clock was instead parametrized with mosaic SoGV and calibrated based on genets of known age. *Acropora palmata* . Despite varying colony size and depth, total somatic genetic variation was consistent within colonies of the same known age class in *Acropora palmata* in Curaçao (ANOVA, *p* > 0.05) (Figure 1b). This indicates that mosaic SoGV feature a linear process of mutation accumulation with age, which allowed us to calibrate a modified somatic molecular clock for *A. palmata* that relies on mosaic and not fixed SoGV. This clock can be applied to any adequately sequenced *Acropora palmata* colony.

Determining the precise distribution of age classes in reef populations of *Acropora palmata* is important for modelling reef futures. Natural sexual recruitment in the Caribbean has been dramatically reduced in recent years (Hughes and Tanner 2000; Roff 2020), calling into question the ability of these populations to adapt to changing conditions. To understand how long this lack of recruitment has been a problem in the population, a reliable method for estimating age in younger colonies is needed and this study is a first step in that direction. Although this method is expensive and carries a high computational effort, given the increasing affordability of whole genome sequencing and availability of computational pipelines, a systematic survey should be designed to estimate the distribution of ages of remaining *Acropora palmata* in the Caribbean.

### 4.2 Stem Cell Demography May Inhibit Mutation Fixation Within Modules

Yu et al. (2024) modelled the allele frequency dynamics of somatic mutations in modular organisms and predicted that the fixation of a mutation in a modular organism such as *Acropora palmata* will be highly reliant on the demography of the stem cell pool. In the seagrass *Zostera marina* , the limited number of apical stem cells (7–12) and a founder cell number of 3–4 allow for a swift and continual fixation with time as the mutations experience a strong bottleneck with the formation of a new ramet. However, in *A. palmata* genets, even in a colony close to a century old, no fixed mutations were detected (Figure 3). This lack of fixation of somatic mutations in old *A. palmata* genets is inconsistent with a module formation process that relies on only a few stem cells. The allele frequency spectra in *Acropora palmata* instead suggest alternative modes of module formation, alone or in combination: a large stem cell pool, multiple stem cell lines, and/or potential movement of cells across the colony that allows for the presence of the same mutation in multiple modules independent of the growth history of the modules.

First, there may be a large stem cell pool. As previously mentioned, models of genetic drift within a modular organism show that stem cell pool (N_e_) and mutation rate are two of the most important factors influencing the allele frequencies of mutations in the population of modules (Yu et al. 2024). Mirroring the population dynamics of whole organisms, increased cell population size during a bottleneck event prolongs the time required for a mutation to achieve fixation within the colony. Therefore, even across many years, the likelihood of fixation would be low in a genet of *Acropora palmata* if the stem cell pool that forms new modules is large.

Alternatively, and not mutually exclusively, stem cells either remain dormant, or somatic cells de‐differentiate into stem cells, which would increase cell population size and effectively perturb any spatial correlation with the pedigree of mutation sharing. The degree of pluripotency of cells across cnidarians is highly variable. Some cnidarians, such as *Hydra*, have three defined ‘stem’ cell lines (Holstein 2023). Other cnidarians demonstrate pluripotency across all stem cell types that facilitate cnidarian's impressive regeneration capabilities. The model cnidarian *Hydra* can regenerate an entire individual from a small population of cells, and the starlet sea anemone *Nematostella* can also regenerate a polyp from a section of tissue (Bossert and Thomsen 2017; Vervoort 2011; Vogg et al. 2019). Regeneration in reef‐building corals is more limited than other cnidarians but is still integral to the health and survival of a colony. When a colony is injured, or experiences partial mortality, the surrounding tissue must recruit cells to regenerate all tissue types and regrow (or ‘reskin’) the injured area (Meesters et al. 1997). Even without prior colony integration, fragments of the same genet, if placed in close enough proximity will grow together to create one integrated colony (Forsman et al. 2015; Page et al. 2018; Highsmith 1982). This regeneration would require the recruitment of stem‐like cells to differentiate and rebuild a whole polyp and all associated tissue and cell types in the colony. Molecular signals of stem‐cells were discovered at the boundaries of wounds where regeneration would occur (Levanoni et al. 2024). Colony integration also allows corals to distribute resources across the entire colony and so repair wounds of any tissue type (Oren et al. 2001). Colony integration may even allow for cells to be shared between modules, and this cell migration may prevent fixation of somatic mutations in subsets of modules in a genet as well as breakdown the expected relationship between genetic and physical distance along the growth axis of a genet.

### 4.3 Evolutionary Consequences of Somatic Mutations in *Acropora palmata*

The fate of somatic mutations in animals and plants can be much more similar than previously thought. In the modular animal, *Acropora palmata* , somatic mutations may not come to fixation and indeed they are often maintained at lower frequency within a genet (this study). Yet, they can still be fixed in the population of *A. palmata* genets when passing through the single‐cell stage of meiotic gamete formation (López‐Nandam et al. 2023; Schweinsberg et al. 2014; Vasquez Kuntz et al. 2022), similar to the inheritance of low frequency somatic mutations in the meiotic offspring of trees (Schmitt et al. 2024).

The significant depletion in mutations found in coding sequences of *A. palmata* (Figure 6) might be an indicator of strong purifying selection within a genet, or of more effective repair systems acting on essential genes, or a mixture of both processes. If true, a high somatic mutation rate is controlled by a high‐fidelity DNA repair mechanism. Mutation rates have been found to scale by lifespan in mammals and not body size (Cagan et al. 2022). Corals represent a unique situation where both lifespan and body size are indeterminate (Bythell et al. 2018). This allows for substantial accumulation of somatic mutations over a coral's lifetime, thus creating a risk of mutational meltdown (Muller's ratchet) (Antolin and Strobeck 1985). The maintenance of mutations in the mosaic state in *Acropora palmata* may allow the genet to generate potentially adaptive genetic diversity (Van Oppen et al. 2011) that can be inherited during sexual reproduction (Vasquez Kuntz et al. 2022), while minimising the mutation burden on the genet as a whole.

The patterns of somatic mutation accumulation documented here do not provide evidence of positive selection for advantageous somatic mutations in *A. palmata*, but they are an important starting point to assess potential selective dynamics under different environmental conditions or in other species, and how much SoGV in the mosaic state could influence phenotype. Moreover, they can predict genet age in a highly clonal coral and reveal stem cell dynamics in a species for which long‐term cell cultures have not successfully been established (Reyes‐Bermudez and Miller 2009). Somatic mutations create a diverse mosaic of novel alleles within a genet and in acroporid corals they can be inherited in gametes, providing additional genetic variants in species where sexual reproduction is limited to once a year (Vasquez Kuntz et al. 2022). This study for the first time analyses the shift in SoGV and the associated allele frequencies of somatic mutations with age in a reef‐building coral and calibrates and applies a somatic molecular clock to determine the age of *Acropora palmata* genet that may ultimately improve demographic and conservation models of populations of *A. palmata*.

## Author Contributions

T.C. performed sample collection, DNA extraction and sample processing, bioinformatics and writing of the paper. J.R. performed modelling of population dynamics within a module of a coral and wrote most of the text describing modelling methods. I.B.B., V.F.C. and Z.D. aided in sample and data collection. B.W., T.B.H.R., and I.B.B. acquired funding. T.C. and I.B.B. conceived the work. T.C. and I.B.B. wrote the paper. All authors edited the paper.

## Funding

This work was supported by Human Frontiers Science Program, RGP0042/2020. National Oceanic and Atmospheric Administration, NA19NMF0080078. Barts Charity Lectureship, MGU045. UKRI Future Leaders Fellowship, MR/V02342X/1.

## Disclosure

*Benefit sharing*: No permitting requirements currently exist to govern access to corals under the Convention of Biological Diversity and its Nagoya Protocol or any other potentially applicable access and benefit sharing laws and regulations of Curacao. Benefits from this research accrue from the sharing of our data and results on public databases as described above.

*Permits*: All research complies with applicable laws on sampling from natural populations and animal experimentation (including the ARRIVE guidelines). All samples were collected in Curaçao under permits provided to CARMABI by the Curaçaoan Government and were imported to the United States under CITES permit 1616US784243/67 where the samples were analysed.

## Conflicts of Interest

The authors declare no conflicts of interest.

## Supporting information


**Figure S1:** Variant allele frequency distributions across age classes. (a) Distribution of variant allele frequencies across all age classes of *Acropora palmata* tested in this study. (b) Distribution of variant allele frequencies within the mosaic range (0.2–0.5).
**Figure S2:** Potential bias introduced through the mutation of methylated gene bodies. (A) Distribution of identified somatic mutations among CpG enriched regions within each genet analysed in this study. CpG islands were identified using the program EMBOSS. (B) Distribution of the proportion of identified somatic mutations among age classes. There was no significant difference in the proportion of mutations identified in CpG islands across age classes.
**Figure S3:** VAF spectra for a single module in a simulated clonal organism with asymmetric cell division and *r* = 5/year. Simulation data (black dots) are normalized so that the first data point has ‘normalized number of variants’ equal to one. Solid yellow line is 1/f^2^ and blue dashed line is 1/f (shifted to pass through the first simulation point). Data for each panel is obtained from 20 simulation runs.
**Figure S4:** VAF spectra for a single module in a simulated clonal organism with asymmetric cell division and *r* = 80/year. Simulation data (black dots) are normalized so that the first data point has ‘normalized number of variants’ equal to one. Solid yellow line is 1/f^2^ and blue dashed line is 1/f (shifted to pass through the first simulation point). Data for each panel is obtained from 20 simulation runs.
**Figure S5:** VAF spectra for a single module in a simulated clonal organism with symmetric cell division and *r* = 5/year. Simulation data (black dots) are normalized so that the first data point has ‘normalized number of variants’ equal to one. Solid yellow line is 1/f^2^ and blue dashed line is 1/f (shifted to pass through the first simulation point). Data for each panel is obtained from 20 simulation runs.
**Figure S6:** VAF spectra for a single module in a simulated clonal organism with asymmetric cell division and *r* = 80/year. Simulation data (black dots) are normalized so that the first data point has ‘normalized number of variants’ equal to one. Solid yellow line is 1/f^2^ and blue dashed line is 1/f (shifted to pass through the first simulation point). Data for each panel is obtained from 20 simulation runs.
**Figure S7:** Distribution of empirical variant allele frequencies across four age classes as compared to the theoretical neutral power law distributions. X‐axes represent the Variant allele frequency (VAF), with increasing VAF towards the right side of the graph. Graphs are presented for each age Class 6, 8, 10 years old and an old colony of unknown age. Theoretical 1/f (blue dotted line) and 1/f^2^ distributions (yellow solid line) are represented in each figure for comparison. All age classes (6, 8, 10) except for the colony of unknown age followed a 1/f distribution at the lower variant allele frequencies.

**Table S1:** Sequencing depth per sample of *Acropora palmata* . Given are the in‐water sample tag and the sample's Baums Lab identification number as well as the colony identification number, sequencing depth and its standard deviation (SD) and the known age of colonies in years. Age of colony P2194 was estimated based on previous microsatellite genotyping.
**Table S2:** Metadata of samples genotyped with the Axiom coral SNP array. Given are the file names (affy_id), the sampling location (Reef), the Baums Lab Identification number, the sample location (tip, branch, or base of the colony), the in water sample tag, and the distance along the branch (cm) where applicable.
**Table S3:** The distribution of allele frequencies of private mutations identified with Mutec2. Given are the sample tag ID, the number of total mutations, the file name of the mutation on zenodo (10.5281/zenodo.15105057), the effective genome size given the bases actually sequenced, the estimated mutation rate (Mutations/Effective Genome Size/Module), the known or estimated age of the colony (in years) and the previously provided mutation rate per year.
**Table S4:** Minimum, maximum and median allele frequencies of mutations by sharing class (private, shared between two or shared between three or four samples per colony) and by age class (6, 8, 10 or > 100 years old).
**Table S5:** Genic mutations. Given is the sample tag number, the total number of mutations in coding regions (genic mutations), the total number of unique mutations regardless of location and the proportion of mutations discovered in genic over the total number of unique mutations as well as the known or estimate age class of the colonies (in years).

## Data Availability

Scripts and associated files are publicly available on Zenodo (https://zenodo.org/uploads/15105057). Scripts used for somatic mutation modelling are available on github (https://github.com/jessierenton/SomaticEvolution.jl). Raw reads are available on NCBI at BioProject PRJNA1244468.
